# Recurrent *Clostridioides difficile* infection worsens anxiety-related patient-reported quality of life

**DOI:** 10.1186/s41687-022-00456-9

**Published:** 2022-05-14

**Authors:** Richard L. Hengel, Claudia P. Schroeder, Jinhee Jo, Timothy E. Ritter, Ramesh V. Nathan, Anne J. Gonzales-Luna, Engels N. Obi, Ryan J. Dillon, Lucinda J. Van Anglen, Kevin W. Garey

**Affiliations:** 1 Atlanta ID Group, 275 Collier Rd, Suite 450, Atlanta, GA 30309 USA; 2 Healix Infusion Therapy, LLC, 14140 Southwest Freeway, Suite 400, Sugar Land, TX 77478 USA; 3 University of Houston College of Pharmacy, 4800 Calhoun Rd, Houston, TX 77004 USA; 4 GI Alliance, 505 S. Nolen Dr, Southlake, TX 76092 USA; 5 Los Robles Health System, 215 W Janss Rd, Thousand Oaks, CA 91360 USA; 6 Merck & Co., Inc, 2000 Galloping Hill Rd, Kenilworth, NJ 07033 USA

**Keywords:** *Clostridioides difficile* infection, Quality of life, Recurrence

## Abstract

**Background:**

*Clostridioides difficile* infection (CDI) is associated with high recurrence rates impacting health-related quality of life (HrQOL). However, patient-reported data are lacking particularly in the outpatient setting. We assessed changes in HrQOL over time in patients treated with bezlotoxumab at US infusion centers and determined clinical factors associated with HrQOL changes.

**Methods:**

The HrQOL survey was conducted in adult patients with CDI, who received bezlotoxumab in 25 US outpatient infusion centers. The survey was adapted from the Cdiff32 instrument to assess anxiety-related changes to HrQOL and completed on the day of infusion (baseline) and at 90 days post bezlotoxumab (follow-up). Demographics, disease history, CDI risk factors, and recurrence of CDI (rCDI) at 90-day follow-up were collected. Changes in HrQOL scores were calculated and outcomes assessed using a multivariable linear regression model with *P* < 0.05 defined as statistically significant.

**Results:**

A total of 144 patients (mean age: 68 ± 15 years, 63% female, median Charlson index: 4, 15.9% rCDI) were included. The overall mean baseline and follow-up HrQOL scores were 26.4 ± 11.5 and 56.4 ± 25.0, respectively. At follow-up, this score was significantly higher for patients who had primary CDI (34.5 ± 21.7) compared to those with multiple rCDI (24.7 ± 21.0; *P* = 0.039). The mean HrQOL change at follow-up was significantly higher for patients without rCDI (34.1 ± 28.8 increase) compared to patients with rCDI (6.7 ± 19.5 increase; *P* < 0.001), indicating improvement in anxiety.

**Conclusions:**

Using the Cdiff32 instrument, we demonstrated that HrQOL worsened significantly in patients with further rCDI. These findings support the use of Cdiff32 in assessing CDI-related humanistic outcomes.

## Background

*Clostridioides difficile* (*C. difficile*), also known as *Clostridium difficile,* is the most common cause of infectious diarrhea in hospitalized patients in the United States (US) [1, 2]. In addition to its negative impact on morbidity and mortality, CDI has been associated with a long-term decline to patients’ health-related quality of life (HrQOL) [3–7]. Studies using quality of life instruments in CDI patients include the Short Form 36-item Health Survey, version 2 (SF-36v2®) [4], the European Quality of life-5 Dimensions-3 Levels of severity (EQ-5D-3L) [5, 6], and the European Quality of life-Visual Analogue Scale (EQ-VAS) [6]. These studies have demonstrated lower HrQOL in CDI patients compared to non-CDI patients. Another study including active and past CDI patients utilized an adapted qualitative survey and reported the majority of participants found their daily activities were impacted, particularly those with active CDI [7].

The *C. difficile* quality of life survey (Cdiff32) was developed to quantify disease-specific changes to HrQOL in patients with CDI [3]. This survey consists of 32 questions, including three domains and five sub-domains, designed to assess CDI-related changes to the physical, mental, and social status of CDI patients. During the development and validation of Cdiff32, the authors determined that the anxiety sub-domain, which is part of the mental domain, was significantly different in patients with primary CDI compared to patients with recurrent CDI (rCDI) [3]. The 2017 Clinical Practice Guidelines for CDI published by the Infectious Diseases Society of America (IDSA) and Society for Healthcare Epidemiology of America (SHEA) have recognized patient-reported outcomes as an important endpoint in clinical research [8]. However, no disease-specific changes to HrQOL over time in patients with CDI have been reported. We used an adapted Cdiff32 instrument to assess HrQOL over time in patients who received bezlotoxumab for prevention of rCDI in outpatient infusion centers. Previously, we reported the use of bezlotoxumab in infusion centers, which provided an opportunity to investigate HrQOL in patients with confirmed CDI [9].

The objective of this study was to assess anxiety-related HrQOL changes in patients with and without rCDI after receiving a one-time infusion of bezlotoxumab in outpatient infusion centers. We examined clinical factors associated with HrQOL changes. Study findings may help support the use of Cdiff32 in assessing impact of new CDI therapeutics on HrQOL changes in CDI patients and increase awareness of HrQOL associated with rCDI.

## Methods

### Study design and data collection

This was a retrospective observational analysis of patient-reported HrQOL data obtained between September 2017 and December 2020 from participating US outpatient infusion centers. Adult patients were included in the study if they received bezlotoxumab and completed the HrQOL survey on the day of bezlotoxumab infusion and again ≥ 90 days post infusion. Completion of both HrQOL surveys was standard procedure for patients receiving a single dose of bezlotoxumab (10 mg/kg) in the outpatient infusion center. Patients were required to have a confirmed positive *C. difficile* test and receive standard of care antibiotic therapy at the time of bezlotoxumab. Baseline demographics, Charlson comorbidity index, prior CDI episodes, CDI risk factors, and rCDI ≥ 90 days post bezlotoxumab were collected from electronic healthcare records. Recurrent CDI was defined as diarrhea lasting ≥ 2 days and resulting in medical intervention with or without a positive stool test for toxigenic *C. difficile*. This study was approved by an institutional review board (IntegReview IRB, Austin, TX, USA).

### Survey instrument

An adapted Cdiff32 survey was used to assess patient-reported HrQOL [3]. In order to construct a survey that could be completed during the patient visit, we included one question from the general physical sub-domain regarding disruption of daily activities and six questions from the anxiety sub-domain. The survey was administered at two different time points and patients ranked their responses on a scale from 1 to 5, with 1 being the least and 5 being the most (Table 1). Patients completed the questionnaire immediately prior to receiving bezlotoxumab (baseline) and again at ≥ 90 days post bezlotoxumab during a follow-up visit or telephone interview (follow-up). Data gathered were de-identified and kept confidential. Response scores were inverted to a scale from 0 (worst HrQOL) to 100 (best HrQOL) using an equal weighting. Changes in HrQOL scores were obtained by subtracting the baseline score from the follow-up score for each patient. A positive value indicated improved HrQOL from baseline.

**Table 1:** *Clostridioides difficile* quality of life survey

No	Sub-domain	Cdiff32 Question #	Question, Text
1	General physical	Cdiff1	Because of your *C. difficile* infection, do you have any difficulties or disruption carrying out your daily activities?
2	Anxiety future	Cdiff5	Are you afraid your *C. difficile* infection could come back again?
3	Anxiety current	Cdiff25	Do you feel irritable because of your *C. difficile* infection?
4	Anxiety current	Cdiff26	Do you feel isolated from others because of your *C. difficile* infection?
5	Anxiety current	Cdiff27	Do you feel depressed because of your *C. difficile* infection?
6	Anxiety current	Cdiff28	Because of your *C. difficile* infection, do you feel life is less enjoyable?
7	Anxiety future	Cdiff29	Do you worry about transmitting your *C. difficile* infection to family and/or friends?

Adapted from *Clostridioides difficile* quality of life survey Cdiff32 [3]

Patient-reported responses were ranked on a scale from 1 to 5, with 1 being the least and 5 being the most

### Statistical analysis

Continuous data were reported as mean and standard deviation (SD) or median and interquartile range (IQR). Categorical data were analyzed as counts and percentages or frequencies. Differences in anxiety-related HrQOL scores in patients who did or did not experience rCDI following bezlotoxumab were stratified by number of CDI episodes and other potentially confounding variables using the Student’s *t*-test. A forward selection, multivariable linear regression model was built to assess changes in HrQOL scores over time controlling for all other clinical variables. Any variable with a *P* value less than 0.05 was considered statistically significant and retained in the model. Statistical analyses were performed using SAS version 9.4 (SAS Institute, Inc., Cary, NC) and IBM SPSS statistics version 22 (IBM, Armonk, NY).

## Results

### Patient characteristics

Overall, 144 patients from 25 outpatient infusion centers were included. Demographics, CDI disease characteristics, and changes in HrQOL scores from baseline to follow-up are shown in Table 2. Mean age was 68 ± 15 years and 63% of patients were female. Median Charlson index was 4 [IQR 3–6]. Prior to receiving bezlotoxumab, 24 patients (16.7%) had primary CDI, 35 (24.3%) had one recurrence, 44 (30.6%) had 2 recurrences, and 41 (28.5%) had ≥ 3 CDI recurrences. Distribution of rCDI risk factors included patients ≥ 65 years of age (*n* = 100, 69.4%), ≥ 1 CDI episode in previous 6 months (*n* = 112, 77.9%), immunocompromised (*n* = 53, 36.8%), current CDI with severe presentation (*n* = 31, 21.5%), use of gastric acid suppressants (*n* = 46, 31.9%), inflammatory bowel disease (*n* = 12, 8.3%), chronic renal disease (*n* = 12, 8.3%), and prior failed fecal microbiota transplant (*n* = 17, 11.8%). At follow-up, 23 patients (15.9%) experienced rCDI within 90 days post bezlotoxumab.

**Table 2:** Changes in health-related quality of life (HrQOL) scores at baseline and follow-up

	No. of patients (%)	HrQOL score (mean ± SD)		Change in HrQOL score
		Baseline	Follow-Up	
*Overall*	144	26.4 ± 11.5	56.1 ± 25.0	29.7
*Demographics*				
Age in years, mean ± SD	68 ± 15			
Female	91 (63.2)	25.9 ± 11.7	54.4 ± 39.5	28.5
Male	53 (36.8)	27.2 ± 12.0	58.9 ± 44.3	31.8
Charlson index, median [IQR]	4 [3–6]			
*CDI history prior to bezlotoxumab*				
Primary CDI	24 (16.7)	34.5 ± 17.1	63.2 ± 46.3	28.7
1 CDI recurrence	35 (24.3)	22.4 ± 8.7	40.0 ± 21.8	17.6
2 CDI recurrences	44 (30.6)	26.9 ± 12.1	60.0 ± 50.1	33.1
≥ 3 CDI recurrences	41 (28.5)	24.3 ± 10.3	53.6 ± 38.5	29.3
*CDI risk factors*				
≥ 65 years of age	100 (69.4)	28.0 ± 12.7	59.1 ± 49.2	31.1
≥ 1 CDI episode in previous 6 months	112 (77.9)	24.2 ± 10.1	55.0 ± 40.3	30.8
Immunocompromised condition^a^	53 (36.8)	20.2 ± 7.2	52.6 ± 37.8	32.4
Gastric acid suppressant use^b^	46 (31.9)	21.2 ± 7.9	50.1 ± 34.6	28.9
Current episode with severe presentation^c^	31 (21.5)	28.1 ± 12.8	63.8 ± 49.1	35.7
Inflammatory bowel disease	12 (8.3)	22.4 ± 8.3	45.5 ± 29.8	23.1
Chronic renal disease	12 (8.3)	31.2 ± 15.5	42.9 ± 27.0	11.7
Prior failed FMT	17 (11.8)	22.7 ± 9.3	56.3 ± 40.1	33.6
*Clinical outcome 90 days post bezlotoxumab*				
rCDI	23 (15.9)	22.0 ± 8.9	28.7 ± 13.5	6.7
No further rCDI	121 (84.1)	27.2 ± 12.0	61.3 ± 46.5	34.1

Patient-reported answers to an adapted *Clostridioides difficile* quality of life survey (Cdiff32) [3] were recorded at baseline (prior to bezlotoxumab) and again at follow-up (90 days post bezlotoxumab) and inverted to a scale between 0 (worst HrQOL) and 100 (best HrQOL)

^a^Due to immunosuppressive medication or underlying disease (immune deficiency, solid organ or hematopoietic stem cell transplant, absolute neutrophil cell count < 500 cells/µL)

^b^Proton pump inhibitor and histamine-2 receptor antagonist

^c^Defined by any of the following: albumin ≤ 3.0 g/dL, serum creatinine ≥ 1.5 times above baseline, hypotension or shock, intensive care unit stay related to CDI, ileus, serum lactate > 5 mmol/L, toxic megacolon or colectomy related to CDI, white blood cell count ≥ 15,000 cells/µL

### Changes in HrQOL scores from baseline to follow-up

The overall HrQOL score averaged 26.4 ± 11.5 at baseline (Table 2). Patients with primary CDI had significantly higher HrQOL scores (34.5 ± 17.1) compared to patients with multiple CDI recurrences (24.7 ± 10.5; *P* = 0.039). However, no differences were observed between baseline HrQOL scores of patients with one CDI recurrence (22.4 ± 8.7) compared to patients with 2 (26.9 ± 12.1) or ≥ 3 CDI recurrences (24.3 ± 10.3). At follow-up, HrQOL scores averaged 56.4 ± 25.0, corresponding to a 2.1-fold increase from baseline score indicating overall improved anxiety-related HrQOL. The mean change in HrQOL score was significantly higher for patients without further rCDI (34.1 ± 28.8 HrQOL increase) compared to patients with rCDI (6.7 ± 19.5 HrQOL increase; *P* < 0.001). Using multivariable analysis, recurrence of CDI post bezlotoxumab (-27.4 ± 6.2 relative change in HrQOL; *P* < 0.0001) and chronic renal failure (− 19.7 ± 8.7 relative change in HrQOL; *P* = 0.025) were significantly associated with reduced changes in HrQOL compared to baseline (− 27.3 ± 6.2 relative change in HrQOL; *P* < 0.0001) (Table 3).

**Table 3:** Univariable and multivariable analyses of changes in health-related quality of life (HrQOL)

Variable	Univariable analysis		Multivariable analysis	
	Relative change in HrQOL score (mean ± SD)	*P* value	Relative change in HrQOL score (mean ± SD)	*P* value
*Patient demographics*				
Female	− 3.3 ± 1.5	0.52		
Charlson index (per 1-unit increase)	1.1 ± 0.97	0.26		
*CDI risk factor present at baseline*				
≥ 65 years	4.6 ± 5.3	0.38		
≥ 1 CDI episode in previous 6 months	− 1.2 ± 6.5	0.85		
Immunocompromised condition^a^	4.2 ± 5.0	0.40		
Gastric acid suppressant use^b^	− 1.2 ± 5.2	0.81		
Current episode with severe presentation^c^	8.7 ± 5.8	0.13		
Inflammatory bowel disease	− 7.2 ± 8.8	0.41		
Chronic renal disease	− 19.7 ± 8.7	0.025*	− 19.5 ± 8.2	0.019
Prior failed FMT	4.4 ± 7.5	0.56		
Recurrence of CDI post bezlotoxumab	− 27.4 ± 6.2	< 0.0001*	− 27.3 ± 6.2	< 0.0001

Results of regression analysis presented as relative mean ± standard deviation given the unit of analysis for HrQOL scores were average changes from baseline. Variables with a *P* value < 0.05 obtained in the univariable analysis were included in the multivariable analysis

^a^Due to immunosuppressive medication or underlying disease (immune deficiency, solid organ or hematopoietic stem cell transplant, absolute neutrophil cell count < 500 cells/µL)

^b^Proton pump inhibitor and histamine-2 receptor antagonist

^c^Defined by any of the following: albumin ≤ 3.0 g/dL, serum creatinine ≥ 1.5 times above baseline, hypotension or shock, intensive care unit stay related to CDI, ileus, serum lactate > 5 mmol/L, toxic megacolon or colectomy related to CDI, white blood cell count ≥ 15,000 cells/µL

## Discussion

Policy makers and clinical practice guidelines recognize patient-reported outcomes as important endpoints in clinical research and practice [8]. Consequently, health-related quality of life (HrQOL) measures seem particularly important in patients with CDI [3, 5, 10]. There are different generic tools created for patient-reported outcomes [11] and quality of life [4–7], however, Cdiff32 is the only instrument specifically developed to assess HrQOL in patients with CDI [3]. A recently conducted real-world experience study of bezlotoxumab in outpatient infusion centers [9] provided an opportunity to assess CDI patient-reported quality of life changes from baseline to follow-up using an adapted Cdiff32 survey. Our findings indicate that patients with primary CDI had significantly higher HrQOL scores at baseline indicating less anxiety compared to patients with multiple prior CDI recurrences. At follow-up, anxiety-related HrQOL worsened significantly in patients with further rCDI regardless of baseline number of CDI episodes, whereas HrQOL improved in patients without rCDI. The findings of HrQOL worsening in patients with further rCDI was confirmed by multivariable analysis.

The strength of this study includes a sizeable cohort as this is the second largest study to date demonstrating quantitative changes of patient-reported HrQOL in CDI [3–6, 10]. In addition, the current study had rigorous individual patient follow-up providing HrQOL data at two different time points. Prospective changes in anxiety-related HrQOL with further episodes of rCDI also suggest this humanistic outcome is important and should be incorporated into future intervention studies designed to prevent rCDI.

Our findings are consistent with previous reports showing decreased HrQOL in patients with CDI recurrences. Han et al. demonstrated low quality of life scores for patients hospitalized with CDI using the Cdiff32 and PROMIS Global Health surveys (10). The Cdiff32 survey was particularly sensitive to HrQOL changes in patients with recurrent and/or severe disease. Others have used various instruments to quantify quality of life in CDI patients [4–7]. Barbut et al. demonstrated the impact of the infection on patients’ quality of life using the EQ-5D-3L and EQ-VAS instruments, which showed an association between HrQOL and CDI severity [6]. Similar results were observed using the EQ-5D-3L questionnaire in patients who were hospitalized with CDI [5]. A multinational study used SF-36v2 to compare the impact of CDI on quality of life and work productivity in patients with current or prior CDI and patients without rCDI [4]. A Canadian study highlighted the long-lasting effect and emotional impact on patients with CDI [12]. All studies have consistently shown decreased HrQOL scores in patients with rCDI, with prevention of rCDI expected to improve HrQOL. The current study findings add to the understanding of the burden associated with CDI and underscore the long-lasting nature of this infectious disease.

The study has limitations. For ease of survey completion, a limited number of questions from the Cdiff32 instrument were used including one question from the general physical sub-domain and six questions from the anxiety sub-domain. Although we were able to demonstrate significant differences between recurrent and non-recurrent CDI patients using this subset of HrQOL questions, future validation of the adapted Cdiff32 survey will be required. This real-world cohort consisted of an older population of CDI patients receiving bezlotoxumab in the outpatient setting. Changes to HrQOL in younger patients and those receiving other treatments will require further research. Nevertheless, the prospective changes of HrQOL scores observed in patients with rCDI suggest that reducing the likelihood of further CDI episodes will likely improve HrQOL. Providing clinically meaningful thresholds that define improvement in HrQOL for patients with CDI should be a focus for future research.

## Conclusions

HrQOL measures are particularly important in patients with CDI,

## Data Availability

The data that support the findings of this study are not publicly available.

## Abbreviations

*C. difficile*: *Clostridioides difficile*
CDI: *Clostridioides difficile* Infection
Cdiff32: *C. difficile* Quality of Life Survey
EQ-5D-3L: European Quality of life-5 Dimensions-3 Levels of severity
EQ-VAS: European Quality of life-Visual Analogue Scale
HrQOL: Health-related quality of life
IDSA: Infectious Diseases Society of America
IRB: Institutional Review Board
rCDI: Recurrent *Clostridioides difficile* infection
SF-36v2: Short Form 36-item Health Survey, version 2
SHEA: Society for Healthcare Epidemiology of America
US: United States
